# Consequences of New Approach to Chemical Stability Tests to Active Pharmaceutical Ingredients

**DOI:** 10.3389/fphar.2016.00017

**Published:** 2016-02-08

**Authors:** Marzena Jamrógiewicz

**Affiliations:** Department of Physical Chemistry, Faculty of Pharmacy with Subfaculty of Laboratory Medicine, Medical University of GdanskGdansk, Poland

**Keywords:** stability, impurities, degradation products, related substances, pharmaceuticals quality

## Abstract

There is a great need of broaden look on stability tests of active pharmaceutical ingredients (APIs) in comparison with current requirements contained in pharmacopeia. By usage of many modern analytical methods the conception of monitoring the changes of APIs during initial stage of their exposure to harmful factors has been developed. New knowledge must be acquired in terms of identification of each degradation products, especially volatile ones. Further research as toxicology prediction during *in silico* studies of determined and identified degradation products is necessary. *In silico* methods are known as computational toxicology or computer-assisted technologies which are used for predicting toxicology of pharmaceutical substances such as impurities or degradation products. This is a specialized software and databases intended to calculate probability of genotoxicity or mutagenicity of these substances through a chemical structure-based screening process and algorithm specific to a given software program. Applying of new analytical approach is proposed as the usage of PAT tools, XRD, HS-SPME GC-MS/MS, LC-MS/MS for stability testing. Described improvements should be taken into account in case of each drug existing already in the market as well as being implemented as new one.

## Introduction

Scientific achievements and innovations play an increasing role in the European economy. “Union of innovation” is one of the seven main initiatives planned within the strategy “Europe 2020” for the creation of an intelligent and balanced economy (European Commission – http://ec.europa.eu/education/policy/strategic-framework/index_en.htm). The concept is mainly to project, develop, produce, and utilize innovative products, industrial processes, and services which are more efficient than those of today. They should effect an improvement in quality of life, as well as help maintain the competitiveness of the European Union (EU) in world markets. This innovation policy is therefore at the nexus of policies concerning research, technological development, and industry.

The EU endeavors to provide society with high quality information about the safety and efficacy of manufactured medicinal products. In turn, the development of pharmaceutical sciences in recent decades has allowed us to obtain reliable data concerning the quality of drugs. The quality of active pharmaceutical ingredients (APIs) must be determined during the development of the conceptual composition and formulation of finished medicinal products, which, besides being therapeutically effective, should also be safe for patients. Evaluation of the quality of APIs is associated with periodic monitoring of the medicinal substance’s state, starting from its synthesis and continuing up to its use in the final medicinal product. The first and pivotal quality parameter, without which the medicinal effects of an API cannot be evaluated, is confirmation of substance’s identity, i.e., the determination of its real chemical structure and its physical form. The purity and stability of an API is also continuously verified. Quality control is one of the most important issues in pharmaceutical studies, in particular in the case of substances that are chemically and physically labile; it is an unceasing challenge for scientists and innovators.

The pharmaceutical quality of APIs is evaluated according to standards and safety requirements developed by many organizations and agencies, including the ICH (International Conference on Harmonization of Technical Requirements for Registration of Pharmaceuticals for Human Use) which was formed in 1991; WHO (World Health Organization), created in 1948; United States FDA (Food and Drug Administration), initiated in 1906; and the EMA (European Medicines Agency), which started its activity 20 years ago as EMEA (European Agency for the Evaluation of Medicinal Products).

## Current Guidelines and Stability Testing

Current guidelines concerning quality evaluation of substances involved in medicinal products concentrate on verification of API stability through determination by particular tests, for which methods are prescribed (ICH Q1A, 1993), as well as on the determination of the presence and quantities of impurities (ICH Q3A, 1994). Researchers investigate different paths of potential degradation of APIs, they evaluate the speed of changes occurring under the influence of different factors, and they assess the possibilities for implementation of stabilizers and ways of protecting labile substances with confirmed therapeutic efficacy. The criteria for management of stability tests as well as the determination and classification of impurities were developed in the early 1990s, but our understanding of the problems of API stability has remained the same since the early 1950s.

The process of evaluation of pharmaceutical stability is composed of different tests, which should determine whether the API is chemically and physically stable. By definition, stability is a state in which the degradation of the API does not occur under the influence of particular factors (Waterman and Adami, 2005). The factors which are recommended by ICH for assessment in stability tests are humidity, temperature, pH, the presence of oxidizing agents, and the influence of light (ICH Q1A, 1993, 2003). The most important stability tests of APIs are so called stress tests, which assume excessive, intense exposure to the action of the factors mentioned above, in individual ways for different APIs. When the chemical stability of APIs is considered, the effects of chemical reactions of the API are evaluated, mainly: hydrolysis in conditions of increased humidity, oxidation in the presence of oxygen or hydrogen peroxide, isomerization, hydration, dimerization, or decarboxylation. Photostability tests are characterized by particular specificity and are an integral part of stability tests that are included in standard ICH Q1A (1993). It is worth noting that the concept of photodegradation is related not only to changes in the structure of the API under the influence of light, but also to the occurrence of free-radical processes, energy transfer, or even luminescence, which may lead to unexpected and atypical results, especially in the solid state (Glass et al., 2004). Although the guidelines were established in 1996, data obtained are still characterized by diversity of the source of irradiation used, as well as by latitude in the irradiation time dependent on the equipment used. There are no guidelines which consider the protection of photolabile substances (Alsante et al., 2014).

The testing of APIs in the environments of high relative humidity, increased temperature, very low or high pH, and exposure to high-intensity electromagnetic radiation from the UV-Vis range may cause significant loss of the API, but above all they initiate the formation of degradation products, which are regulated by other guidelines concerning impurities. An advantage of stress tests is that they determine the reactivity of the substance analyzed, as well as the mechanism of its degradation, which provides valuable information used in the succeeding stages of drug formulation technology, especially during manufacturing. These data may also be used to protect the environment around pharmaceutical factories, for example, photodegradation processes may be used for the protection of groundwater. It is worth noting that stress tests may lead to the formation of different products depending on the stage of the degradation reaction, similar to the case during free-radical reactions where the phases initiation, propagation and termination are significantly different. Therefore, it is very important to monitor the state of an API and the products formed over different time intervals. Most commonly, stress tests establish from 5 to 30% loss of APIs (Reynolds et al., 2002; Alsante et al., 2014).

Another issue connected with the evaluation of API stability is physical stability. During stability tests, physical changes in substances may occur, such as change of polymorphic form (Yoshioka and Aso, 2007), mobility of an amorphous form (Chieng et al., 2011), or phase transition (Brittain et al., 1991).

Where an API is unstable in stress tests, degradation products are formed, which are included in the group of so called “specific impurities” of the API. According to ICH, an impurity is any component of the API which is not structurally or physically defined as that particular API and causes distortion of its purity. Impurities are divided into three types which are regulated by different guidelines from ICH and pharmacopeias (ICH Q3A, 1994; European Pharmacopoeia, 2015): the group of organic impurities consists of substrates from the API synthesis, intermediates, degradation products, reagents, ligands, and catalysts; the group of inorganic impurities consists of reagents, ligands, catalysts, heavy metals, inorganic salts, and others, such as carbon; residual solvents fall within the third group.

## Low Concentrations of Impurities Must Be Identified *In Silico*

The determination of impurities in APIs is a consequence of testing their chemical stability and relates to knowledge of structure, physicochemical properties, the assay and toxicology (Carstensen, 1999; Olsen and Larew, 2005; Huynh-Ba, 2008). The first ICH regulations concerning quantitation limits of impurities in APIs (ICH Q3A, 1994) were based on the daily dose of the API taken by the patient, the route(s) of administration and the time of therapy; they did not anticipate the need for impurity identification at concentrations <0.1% (ICH Q3B, 2000). In most cases the finished drug product was used for the quantitative evaluation of impurities, rather than the API itself. In 2004, a significant change in approach took place. Apart from lowering the acceptable limits of quantitation of impurities in APIs and medicinal products which had been developed earlier by EMEA, action was taken to study their toxicity in each situation where they are detected in a drug to estimate their genotoxic properties (EMEA, 2004). Emphasis was laid on dangerous chemicals that interact with DNA, such as *N*-oxides, aromatic rings, aromatic amines, aldehydes, aromatic nitric groups, and alkyl-*N*-nitrosamines (McGovern and Jacobson-Kram, 2006). For example, impurities that have undergone stricter evaluation are hydrazine derivatives associated with the medicinal compounds mildronate and celecoxib. The degradation product 1,1,1-trimethylhydrazinum bromine was determined using very specific methods, i.e., HILIC (Liu et al., 1996) or HS-GC-ECD (Sun et al., 2010). Alkyl halides are another equally toxic group of compounds and are often volatile; they are particularly monitored in drug substances such as diltiazem or azasetron where the impurities are the highly mutagenic benzyl chloride, chloroethyl methyl ether, and *N,N*-dimethylaminoethyl chloride (Reddy et al., 2015). Benzene derivatives such as (*S*)-4-nitrophenylalanine hydrate and (*S*)-methyl-4-nitrophenylalanate hydrochloride, which were found as impurities of zolmitriptan, have been confirmed to undergo DNA damaging reactions using UHPLC-MS/MS and LC-MS/MS. Recently, research demonstrated that a GC technique coupled with HS-SPME (head-space solid phase microextraction) and a MS detector allowed the detection and identification of volatile photodegradation products of ranitidine hydrochloride, such as acetaldoxime, thiazole, dimethylacetamide, dimethylformamide, and 5-methylfurfural (Jamrógiewicz and Wielgomas, 2013), which have hepatotoxic, carcinogenic, and genotoxic properties.

To minimize risk, the recent ICH M7 guidelines (2014) adopted as a requirement in stability testing the use of at least two *in silico* tools for prediction of toxicity, genotoxicity, carcinogenicity, and allergenicity on the basis of chemical structure and specialized knowledge. Some computer techniques have been accepted for use to meet such requirements. There are specialized pieces of software and databases intended to calculate the probable toxicity of substances through chemical structure-based screening processes and software specific algorithms. It has been proposed that the lowest possible quantitation limit of impurities should be enforced in cases of the predicted presence of a genotoxic substance formed from an API (Dow et al., 2013). Generation of new criteria concerning impurities is a great challenge, because they make it necessary to determine in a precise way the rate of formation of degradation products in a genotoxic class from the guidelines Q3A and Q3B (ICH M7, 2014) at levels <0.1%. Examples of *in silico* tools are QSAR (quantitative structure-activity relationships), which enables the estimation of the connection between a structure, the properties resulting from it and the toxic activity of the chemical substance, and DEREK (deductive estimation of risk from existing knowledge).

## Older Generation APIs – The Need For New Stability Studies

Changes to guidelines implemented in the last decade indicate that the monitoring of structural changes should be conducted not only for new APIs, but also that older medicinal substances should be under constant control and monitoring (Olsen and Larew, 2005). Changes connected with quality control of APIs are a chance to identify atypical and dangerous impurities of an API, as well as for the development of methods to eliminate them (Alsante et al., 2014; Görög, 2015). Review of older APIs is concerned with the fact that despite the great quantity of research and stress tests that were performed on these APIs, the data become incomplete in the light of new guidelines. Moreover, the development of analytical methods broadens the possibility for identification of degradation products of APIs that were not observed before. Therefore, there is a need to reinvestigate instability effects observed previously and to evaluate the toxicity of the impurities determined. Both EMA and WHO underline that nowadays there is widespread access to analytical tools which enable assessment of APIs to reach the quantitation limits described in guidelines. Some available analytical methods, such as liquid/gas chromatography (LC/GC), are characterized by great specificity and sensitivity, so enable the determination and identification of substances at a very low level (McGovern and Jacobson-Kram, 2006; Giordani et al., 2011; EMA, 2012; WHO, 2012; Görög, 2015).

## Protection of Chemically Labile APIs

Where possible, it is worth protecting APIs from harmful factors to prevent molecular degeneration. Chemical and physical knowledge obtained over many years allow us to use different systems which may protect APIs to avoid their degradation, preventing the formation of impurities. For example, from 1980 to 1990 the protective properties of cyclodextrins and other supramolecular systems were discovered, and in recent years many papers have discussed nanotechnological methods of API stabilization (Baek et al., 2014). Suitable guidelines concerning this issue have not yet been developed, probably because there is great heterogeneity in the properties of labile molecules, but conclusions about the measurable benefits of physical and chemical protection of APIs can be drawn from the papers of many researchers.

## Process Analytical Technology in Stability Testing of APIs at the Earliest Time Possible

In 2002, the FDA took action in the area of quality control as well as the evaluation of pharmaceutical stability. This concerned all manufacturing steps in which contact between an API and a potentially harmful factor was possible and in consequence the formation of degradation products may occur. From then on, efforts were concentrated on quality evaluation through the assurance of reliability in monitoring the technological processes, taking into consideration their variability and their influence on the quality attributes of APIs. Procedures were collected in one general initiative, “*Process Analytical Technology*” (PAT; FDA, 2003), which covers integrated chemical, physical, microbiological and mathematical analysis as well as risk analysis of quality loss. Specialized systems were implemented for control measurements of the state of APIs to inform on stability. However, PAT lacks a requirement to achieve full identification of degradation products (i.e., impurities according to Pharmacopoeias) that are produced. Based on FDA documentation, pharmacopeial documentation, and ICH guidelines related to process control of APIs (ICH Q9, 2005; ICH Q10, 2008; ICH Q8, 2009), EMEA (2009) proposed using analytical tools which are fast, non-destructive alternatives to separation methods such as chromatographic techniques, that allow assessment of the quality, identity, stability, and purity of samples containing APIs. Additionally, it was stressed that traditional analytical methods are no longer sufficient, mainly due to the inability to study real-time processes. For this purpose PAT recommends the use of near-infrared spectroscopy (NIR; EMEA, 2009) and Raman spectroscopy, which enable the management of qualitative and quantitative analysis not only in real manufacturing mode, so *on-line*, but also in laboratory mode, *at-line*, and remotely, *in-line*. Moreover, methods supporting PAT, e.g., for the direct evaluation of stability, were indicated; these methods allow monitoring of potential changes occurring in solid samples, as well as in some isolated or synthesized liquid bulk materials, and in biosynthesized materials such as enzymes and peptides (without dissolution of the samples).

Roentgenographic methods were included in the group of tools supporting PAT, for example X-ray diffraction (XRD), as well as thermoanalytical methods (e.g., differential scanning calorimetry (DSC), thermogravimetric analysis), and microscopy (atomic force microscopy, scanning electron microscopy). In pharmaceutical technology the most important issue is to confirm the crystallographic structure, or to assess the spatial conformation of synthesized APIs and excipients, by means of roentgenographic techniques (Paradowska and Wawer, 2014), but this became common only recently. On the basis of the so called “thermal history” of the sample analyzed, information about the API’s quality may be obtained by means of differential scanning calorimetry and thermogravimetry. These tools have great analytical potential for quality evaluation, as well as for monitoring the state of APIs. Similarly, microscopic techniques, in connection with spectroscopic techniques like Raman spectroscopy or fluorescence spectroscopy, with the possibility of chemical imaging, are useful tools supporting PAT conceptions.

It can be concluded that API stability assessment is not only still valid, but expanding in scope. It is important that pharmaceutical literature emphasizes the need to conduct such research into new medicinal substances, as well as older generation products (Olsen and Larew, 2005) where studies were conducted according to older guidelines using out-of-date methods.

**FIGURE 1:**
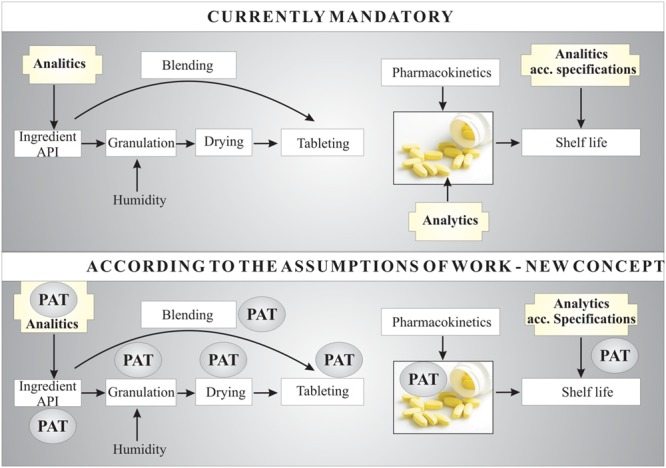
**Scheme of the new conceptual approach to chemical stability tests of APIs – in this example, applied to tablets**.

One reason that new concepts for stability testing should be considered (**Figure 1**) is the lack of clear guidelines on the quality estimation procedure for therapeutic substances after a short period of exposure to a damaging factor. Direct stability studies of APIs in the solid, semi-solid or liquid phase, particularly in the short term, are a new concept for monitoring API quality. The first steps in degradation of an API compound are initiation (such as photodegradation) and propagation; these are dynamic processes that can lead to a number of degradation pathways of the API molecule. Moreover, unknown (or previously unrecognized) degradation compounds produced can be dangerous to humans (e.g., cytotoxic, genotoxic, or mutagenic). For example, the scientific literature and pharmacopeial guidelines do not mention that attention should be paid to the production of volatile degradation products of APIs because of their own toxicity profiles. Particular databases should be developed for each type of preparation or drug. PAT, as a quality control system including measurement of stability at the various stages of manufacture of a medicinal product, fulfills its role for each type of preparation or drug (orally administrated, injections, and others). However, broadly considering new guidelines aimed toward the prevention of toxic degradation products, PAT is not sufficient. Some additional analytical procedures should be included in stability tests wherever there is any information about changes in the structure of a molecule resulting from its sensitivity to any damaging agents. At the first sign of changes, the resultant impurities should be researched, their toxicity assessed and action taken to eliminate them.

To achieve positive, significant improvement of stability testing, the most important step is to implement a new pathway to determine the safety of drug substances:

(a) testing of solid, semi-solid or liquid samples of medicinal substances in terms of exposure to degradation factors such as high temperature, humidity, and light;
(b) evaluation of changes on the basis of optimized spectral analysis by FTIR/NIR spectroscopy, DSC, XRD patterns, and chromatograms from GC-MS and LC-MS/MS;
(c) determination of time wherein the labile drug substance does not change physically or chemically under the conditions of exposure;
(d) monitoring for the presence of volatile degradation products in the samples;
(e) quantitative assessment of ongoing changes in the drug directly in raw materials;
(f) preliminary assessment of the usefulness of the results obtained compared to available and applicable pharmacopeial procedures;
(g) initial assessment of patients’ exposure to selected degradation products of medicinal substances that may be cytotoxic (*in silico* modeling);
(h) stability testing of the medicinal product including sensitive APIs (with toxic degradation compound profiles) according to the above proposed procedure;
(i) protection and prevention of API degradation in medicinal products in case of a confirmed degradation profile (for example, by improved coating, use of capsules vs. tablets or inclusive complex formation).

**FIGURE 2:**
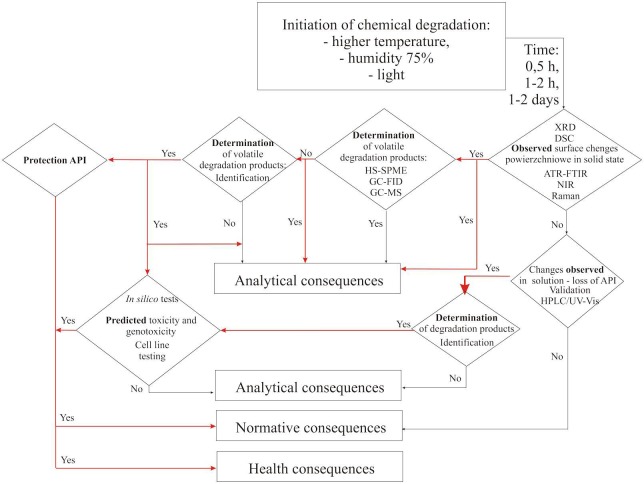
**New analytical workflow concerning different consequences of stability testing of APIs**.

It is worth noting that this new analytical procedure, including the involvement of different techniques and procedures for the monitoring of stability of APIs, will allow users to cope with the most difficult research aims and will influence the normative consequences (**Figure 2**). The obtaining of information about changes in the structure of an API under the influence of destructive factors will became a signal for the creation of new standards. The aim of evaluation of normative records is to show permissible levels of newly identified compounds, as well as to broaden the range of research. The most important normative consequence is the possibility of publishing a decision about withdrawal of a particular API, even one which has been used in therapy, based on new data which are important from the safety-of-drugs point of view.

Determination of impurities that are described as pharmacopeial, on which most analytical procedures for the quality control of APIs and drugs are based, is insufficient in the light of new requirements. Most importantly, there is a lack of practical guidelines that indicate how to deal with determination of specific impurities, including those that are on the list of the most dangerous compounds with potential genotoxic activity. Published studies clearly indicate the need to focus on procedures for the detection, identification, and elimination of genotoxic substances present simultaneously with APIs in medicinal products. The first observations of change must be made on a short timescale (e.g., 0.5 h), for example in raw material, using validated PAT tools; then, other methods should be involved to confirm the presence of and quantify compounds hazardous to the health of patients. Finally, all activities should aim to eliminate genotoxic impurities and/or protect the API against degradation. There is a need to monitor impurities from all ICH classification groups, with special attention paid to volatile substances. Moreover, the collection and preparation of samples, especially non-standard ones, should be covered by obligatory procedures involving modern analytical tools. The monitoring of the state of a molecule in different stages of degradation, occurring because of different chemical reactions, is innovative and has not been practiced in the past. Exhaustive data about the chemistry of APIs may be obtained only by carrying out complementary research. Nowadays, it is a priority to determine the toxicology of a compound so as to ensure pharmaceutical quality of the drug in a way that is compatible with the appropriate standards.

Highlights of this project is also additional aspect, has not been featured in literature or in FDA or WHO regulations, which has become a primary objective of proposed research (Jamrógiewicz and Ciesielski, 2015).

## Author Contributions

The author conceived and designed the work, wrote the paper, performed the bibliographic research and edited the manuscript.

## Conflict of Interest Statement

The author declares that the research was conducted in the absence of any commercial or financial relationships that could be construed as a potential conflict of interest.
